# SEX DIFFERENCES IN THE ASSOCIATION BETWEEN BODY COMPOSITION AND 2-YEAR CHANGE IN COGNITIVE FUNCTION

**DOI:** 10.1093/geroni/igac059.2271

**Published:** 2022-12-20

**Authors:** Daehyun Lee, Miji Kim, Hyung Eun Shin, Jae Young Jang, Chang Won Won

**Affiliations:** Kyung Hee Univerisity, Seoul, Seoul-t'ukpyolsi, Republic of Korea; Kyung Hee University, Seoul, Seoul-t'ukpyolsi, Republic of Korea; Kyung Hee University, Seoul, Seoul-t'ukpyolsi, Republic of Korea; Kyung Hee University, Seoul, Seoul-t'ukpyolsi, Republic of Korea; Kyung Hee University, Seoul, Seoul-t'ukpyolsi, Republic of Korea

## Abstract

In the process of aging, the loss of lean mass and increase in fat mass are associated with cognitive decline. This study investigated sex differences in the association between body composition and changes in cognitive function in community-dwelling older adults in Korea. A total of 1,420 participants (aged 70–84 years, 54.2% men) of the Korean Frailty and Aging Cohort Study with data from baseline and 2-year follow-up surveys were included. Body composition was measured using dual-energy X-ray absorptiometry and cognitive function was assessed using the Korean version of the Consortium to Establish a Registry for Alzheimer’s Disease Assessment Packet. The total fat mass was lower in men than in women (p< 0.001), whereas total lean mass was higher in men than in women (p< 0.001). Total body fat mass was positively associated with the time taken to finish the Trail Making Test-A only in women (standardized beta coefficient [ß]= -1.371, p=0.018), and a negative association was observed between trunk fat mass and digit span total only in men (ß= -0.092, p=0.039). Appendicular lean mass was significantly positively associated with word list recognition only in women (ß=0.087, p=0.010) and was significantly positively associated with digit span total (ß=0.108, p=0.027) and digit span forward (ß=0.081, p=0.025) only in men. The results of this study indicated that higher fat mass was associated with the protection of decline in cognitive function only in women, while lean mass was positively associated with a change in cognitive function in both sexes.

